# Colloidal dispersion of poly(ionic liquid)/Cu composite particles for protective surface coating against SAR‐CoV‐2

**DOI:** 10.1002/nano.202100069

**Published:** 2021-06-01

**Authors:** Atefeh Khorsand Kheirabad, Xuefeng Pan, Siwen Long, Zdravko Kochovski, Shiqi Zhou, Yan Lu, Gerald McInerney, Jiayin Yuan

**Affiliations:** ^1^ Department of Materials and Environmental Chemistry (MMK) Stockholm University Stockholm Sweden; ^2^ Department for Electrochemical Energy Storage Helmholtz‐Zentrum Berlin für Materialien und Energie Hahn‐Meitner‐Platz 1 Berlin Germany; ^3^ Department of Microbiology Tumor and Cell Biology Karolinska Institutet Stockholm Sweden; ^4^ Institute of Chemistry University of Potsdam Potsdam Germany

**Keywords:** antivirus coating, colloidal dispersion, copper nanoparticle, poly(ionic liquid), SARS‐CoV‐2

## Abstract

Herein, we report a waterproof anti‐SARS‐CoV‐2 protective film prepared by spray‐coating of an aqueous colloidal dispersion of poly(ionic liquid)/copper (PIL/Cu) composite nanoparticles onto a substrate. The PIL dispersion was prepared by suspension polymerization of 3‐dodecyl‐1‐vinylimdiazolium bromide in water at 70°C. The copper acetate salt was added into the PIL nanoparticle dispersion and in situ reduced into copper nanoparticles anchoring onto the PIL nanoparticles. Despite being waterborne, the PIL in bulk is intrinsically insoluble in water and the formed coating is stable in water. The formed surface coating by PIL/copper composite nanoparticles was able to deactivate SARS‐CoV‐2 virions by 90.0% in 30 minutes and thus may effectively prevent the spread of SARS‐CoV‐2 through surface contact. This method may provide waterborne dispersions for a broad range of antivirus protective surface coatings for both outdoor and indoor applications.

## Graphical abstract

A waterproof anti‐SARS‐CoV‐2 protective film was prepared by spray‐coating of an aqueous colloidal dispersion of poly(ionic liquid) /copper (PIL/Cu) composite nanoparticles onto a substrate. The formed surface coating by PIL/copper composite nanoparticles was able to deactivate SARS‐CoV‐2 virions by 90.0% in 30 minutes and thus may effectively prevent the spread of SARS‐CoV‐2 through surface contact.

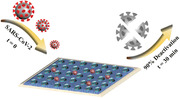

## 1 INTRODUCTION

The emergence of a current pandemic, caused by a novel coronavirus, severe acute respiratory syndrome coronavirus type 2 (SARS‐CoV‐2), has brought a rapid viral infection over 200 + countries and more than three million deaths globally by the time of this report. The transmission from infected to healthy persons can be transferred through cough, respiratory droplets, biofluids or close contacts. These respiratory pathogens can remain infectious on insentient surfaces for hours to 2‐3 days.^[^ ^1^, ^2^ ^]^ Consequently, the determination of antiviral coating materials with high antiviral activity and broad applicability on various surfaces may eliminate the possible infection transfers and reduce the speed of virus spread.^[^ ^3^, ^4^, ^5^ ^]^

Metal nanoparticles historically have been proven to show a wide range of virucidal and bactericidal activities due to their effectiveness at a low dosage, small size, high surface area, and acting as ion reservoirs to control the release of bioactive ions. Metal nanoparticles demonstrate the capability to generate reactive oxygen species to destroy the virus and attach to DNA or RNA, and accordingly they can prevent the replication of microorganisms. Since past decades, various types of metal nanoparticles, such as silver (Ag), copper (Cu), iron, iron oxide, gold, and titanium oxide, with antibacterial and virucidal activity have been described vastly in literatures. Among those, Ag nanoparticles are the most widely explored in the literature because of their high effectivity versus infections and high demands in industrial applications. Regardless of multitude benefits of Ag nanoparticles, Cu nanoparticles with high antimicrobial (especially antifungal), low cost and wide availability and abundance can be alternatively utilized.^[^ ^6^, ^7^, ^8^, ^9^, ^10^, ^11^ ^]^ If not surface‐protected, a rapid aggregation of a dispersion of native metal nanoparticles can cause a large decrease in their reactivity. A common method to overcome this problem is to stabilize metal nanoparticles with organic compounds, such as low molecular weight ligands and chelating polymers by selective functionalization of their surface.^[^ ^12^, ^13^, ^14^, ^15^, ^16^ ^]^

Poly(ionic liquid)s (PILs) as a subclass of polyelectrolytes are polymer materials with adjustable physiochemical characteristics due to covalently incorporated ionic liquid‐like ion pairs in the polymer chains, and usually possess mechanical stability, durability, ion conductivity and multifunctionality.^[^ ^17^, ^18^ ^]^ This unique combination broadens the application scope of PILs in comparison to conventional polyelectrolytes in versatile research areas such as biosensors,^[^ ^19^, ^20^ ^]^ biomaterials,^[^ ^21^, ^22^, ^23^, ^24^, ^25^ ^]^ energy materials,^[^ ^26^, ^27^, ^28^, ^29^ ^]^ just to name a few. Besides, the self‐assembly of PILs has provided new aspects in the direction of functional nanomaterials.^[^ ^30^, ^31^, ^32^, ^33^ ^]^ Thus, it is our opinion that well‐defined self‐assembled PIL nanoparticles can be promising candidates to support and stabilize metal nanoparticles to maintain metal's antiviral and antibacterial activity.^[^ ^34^, ^35^ ^]^ Previously our group investigated straightforward self‐assembly of imidazolium and triazolium‐based PIL nanoparticles with different alkyl chain lengths and tunable structures. These PILs due to the presence of long alkyl chain can self‐assemble into multi and unilamellar vesicles, or wasp‐like dispersible nanoparticles when they are produced in water^[^ ^36^, ^37^, ^38^ ^]^ although these PILs in bulk are water‐insoluble.

In this contribution, we investigated the potential anti‐SARS‐CoV‐2 activity of a composite film consisting of spray‐coated PIL/Cu nanoparticles from their aqueous colloidal dispersion. Detailed materials characterization certified the establishment of smaller Cu nanoparticles onto the PIL nanoparticles. Our virucidal activity tests show high virucidal activities of such films against SARS‐CoV‐2 virions, pointing out potential applications of such spray‐coatings for indoor and outdoor surfaces to decline the infection and transmission of SARS‐CoV‐2 virions *via* contaminated surfaces.

## 2 RESULTS AND DISCUSSION

**FIGURE 1:**
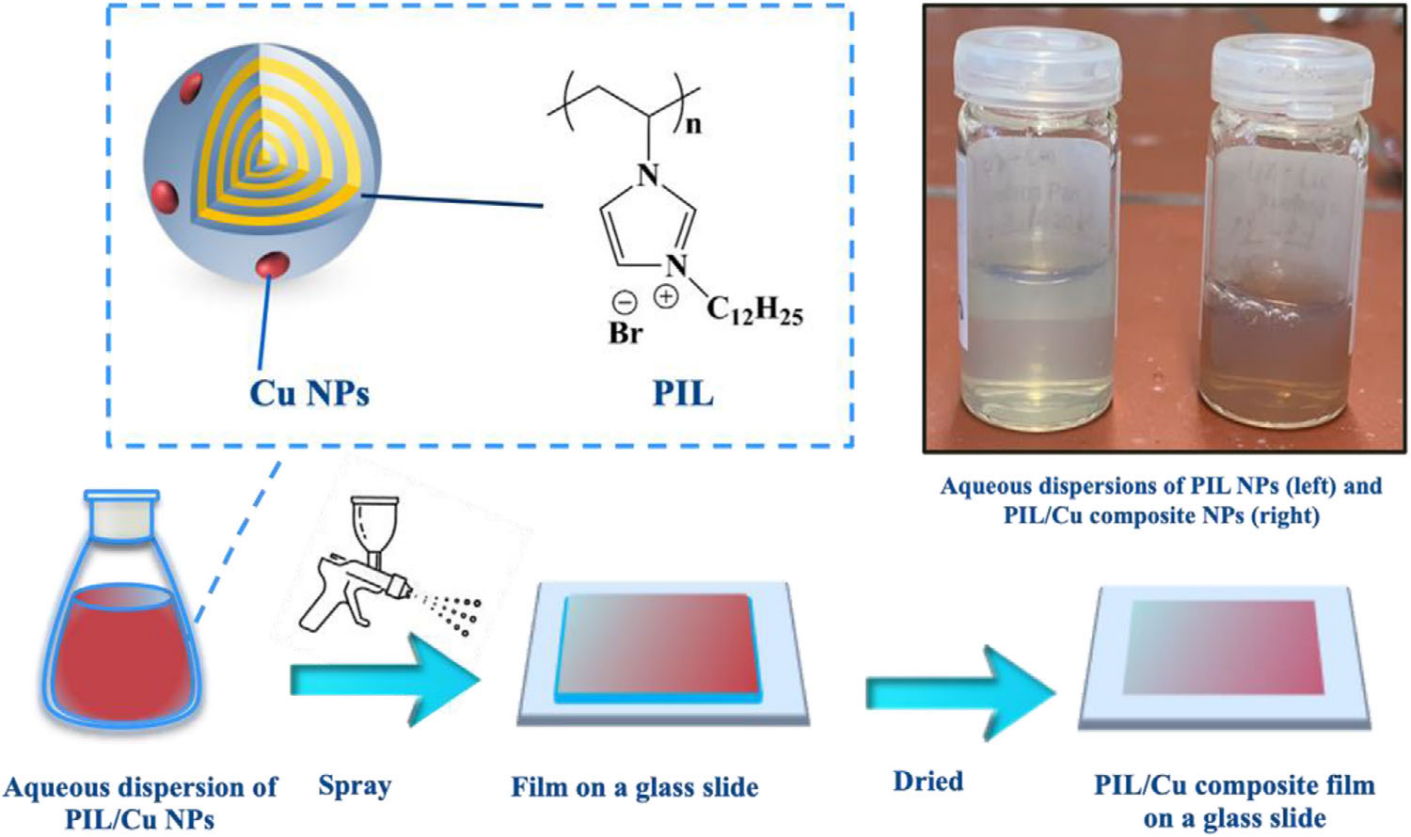
Schematic representation of the surface spray coating of a glass substrate by an aqueous colloidal dispersion of PIL/Cu composite nanoparticles

The stepwise fabrication of the spray‐coating film onto a glass slide with a colloidal dispersion of PIL/Cu composite nanoparticles is provided in Figure 1. The colloidal nanoparticle dispersion comprises a composite of PIL nanoparticles containing Cu nanoparticles that are located on PIL nanoparticles. The synthesis and characterization details of the PIL used in this study, named poly(1‐*n*‐dodecyl‐3‐vinylimidazolium bromide) are provided in Supporting Information.

The convenient method to obtain Cu nanoparticles in the solution phase is the chemical reduction of a copper salt by a proper reducing agent. Several reducing agents can be utilized at different reaction temperature, for example, sodium borohydride at room temperature, hydrazine (N_2_H_4_, at 80°C), and ascorbic acid (vitamin C, at 100°C).^[^ ^39^, ^40^, ^41^ ^]^ Depending on the choice of the reducing agent, water‐soluble by‐products may be generated after the reduction of copper salt. An additional step of dip‐washing of the film product in clean water for 30 seconds is added to remove any by‐product.

In a typical surface coating procedure, copper acetate was added into the aqueous dispersion of PIL nanoparticles. In following, a reductant hydrazine (N_2_H_4_) was added at 80°C to reduce the Cu ions into Cu nanoparticles, which are stabilized in situ by the surface of PIL nanoparticles. To ensure the full reduction of metal salts to metal nanoparticles, the reducing agent was added in a two molar equivalent ratio to the metal salt. Next, via a spray setup, the dispersion solution was sprayed onto the surface of a glass slide, which due to the water‐insolubility of the PIL forms a waterproof film that remains intact when dipped washed in water. The film on the glass slide was then placed under vacuum (5 × 10^−2^ mbar) and fully dried in an oven at 80°C at least for 2 hours. More detailed information about the coating preparation method is provided in Supporting Information.

**FIGURE 2:**
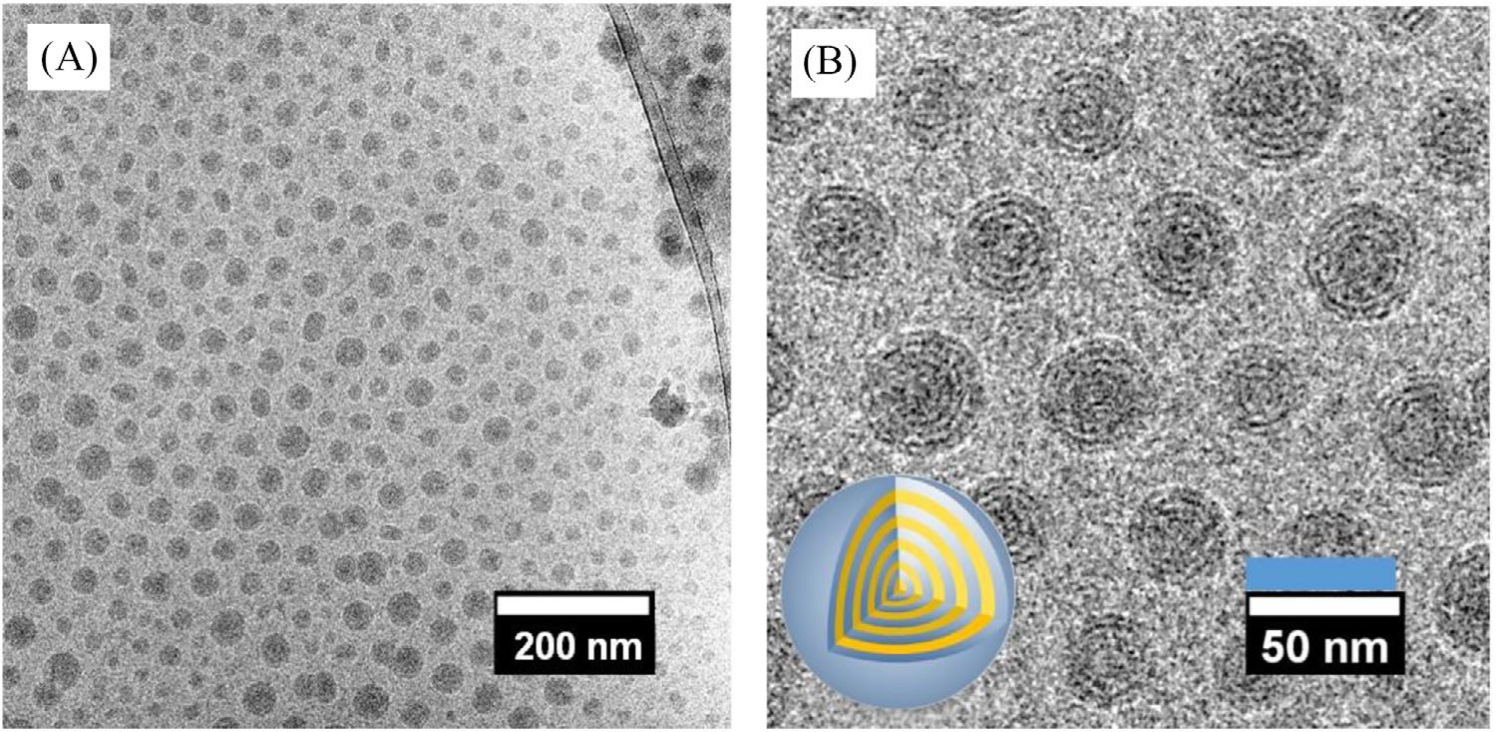
A), Cryo‐TEM overview image of the pristine PIL nanoparticles dispersed in aqueous solution. B), The enlarged view (The inset on the bottom left is a cartoon showing the inner structure of a single PIL nanoparticle)

In Figure 2A and  2B, cryogenic transmission electron microscopy (cryo‐TEM) images of the PIL nanoparticles with a long dodecyl substituent on the vinylimidazolium repeating unit in aqueous solution are provided. The dark dots represent the PIL nanoparticles in a spherical shape. The native inner morphology is clearly distinguishable, where a quasi‐spherical onion‐like vesicular nanostructure is identified with its size variation in the range of 20–50 nm. The dark ring within the nanoparticles represents the ionic mainchain domain, and the higher contrast originates from the Br^−^ anion that serves also as a contract agent here. The grey ring zones come from the packed alkyl substituents that are of a lower contract in the TEM. The number of bilayers of ca. 3.42 nm in thickness is countable in the typical range of 4–8 and is dependent on the size of nanoparticles. Previously, the analysis of small‐angle X‐ray scattering (SAXS) measurements showed a *d* spacing of 3.40 nm for PIL nanoparticles with a dodecyl substituent,^[^ ^38^ ^]^ which is in agreement with the value determined here by the cryo‐TEM tests.

**FIGURE 3:**
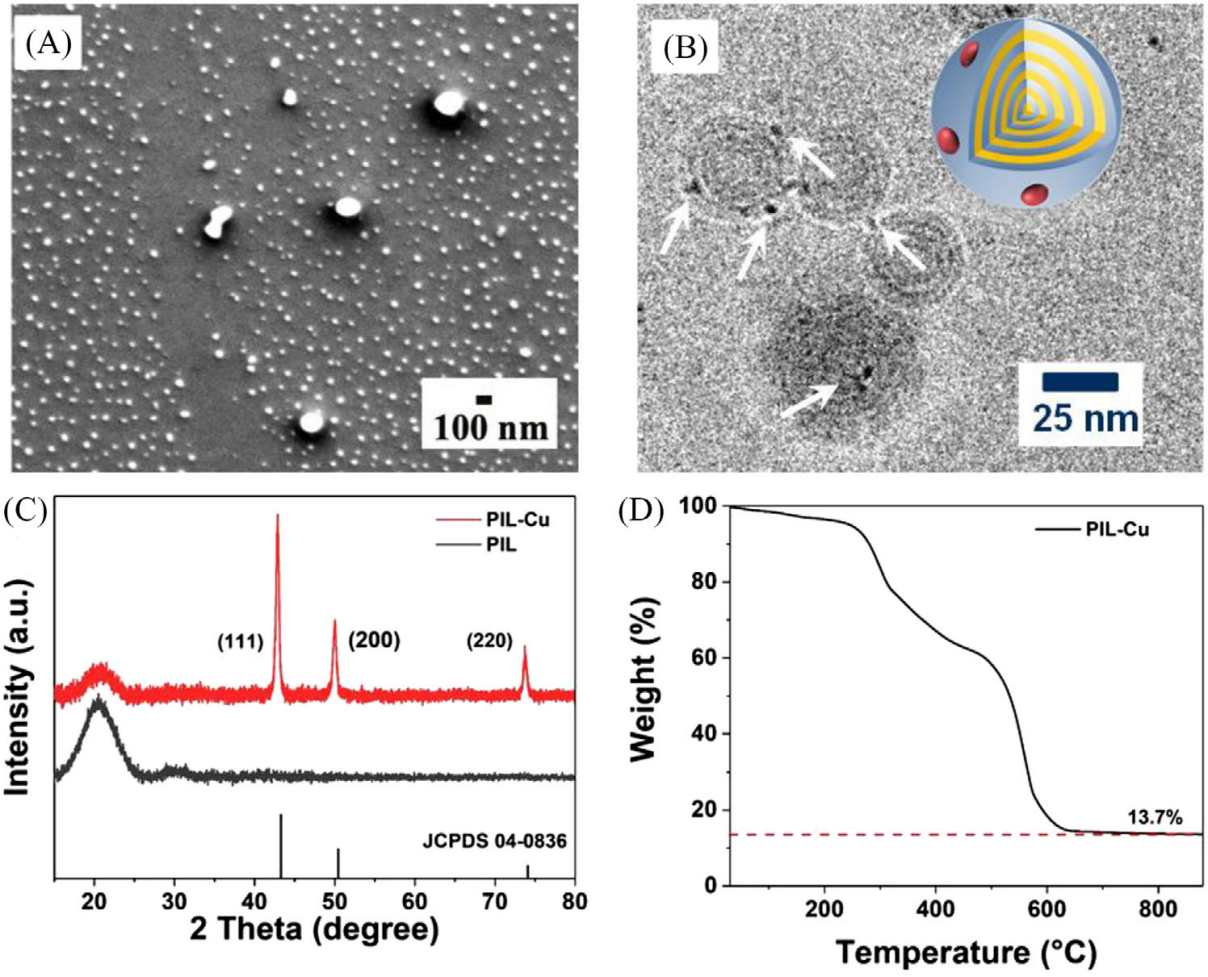
A), SEM image of the PIL/Cu composite nanoparticles on a glass substrate. B), Cryo‐TEM image of PIL/Cu nanoparticles dispersed in aqueous media. C), XRD patterns of PIL and the PIL/Cu composite nanoparticles. D), TGA curve of the PIL/Cu composite nanoparticles under air from room temperature to 900 °C

The scanning electron microscopy (SEM) shown in Figure 3A visualizes a composite of PIL/Cu colloidal nanoparticles in a spherical shape with a similar size as the pristine PIL nanoparticles. The sample morphology on the glass substrate does not change after the glass plate was immersed in water for 10 minutes, a phenomenon that arises from the insoluble nature of the PIL nanoparticle in water and its strong surface‐binding function. But the presence of Cu nanoparticles cannot be demonstrated by SEM due to their rather small size. Therefore, cryo‐TEM for the PIL/Cu colloidal composite was employed (Figure 3B). Clearly, Cu nanoparticles of 1–4 nm in size are observed to locate on PIL nanoparticles, highlighted by white arrows. It is worthwhile to mention that particles smaller than 1 nm may exist but are not observable due to the weak contrast between the PIL matrix and the Cu nanoparticles in the cryo‐TEM image. In order to confirm the existence of Cu nanoparticles in the composite dispersion, X‐ray diffraction (XRD) patterns of the dried PIL nanoparticles and PIL/Cu composite were recorded and are compared in Figure 3C. The comparison between these two patterns reveals that the PIL nanoparticles are obviously in an amorphous state, and the presence of all three sharp peaks in the XRD pattern of the PIL‐Cu composite sample is in accordance with the standard pattern of pure face‐center cubic (FCC) metallic Cu (JCPDS, File No 04–0836); the three peaks at 43^o^, 55^o^, and 74° correspond to the (111), (200), and (220) reflections.^[^ ^42^, ^43^ ^]^ In Figure 3D, the thermogravimetric analysis (TGA) of the PIL/Cu colloidal composite in air shows a 5 wt.% mass loss at 250°C, and then a rapid decomposition up to 600°C, followed by a constant mass loss till a final residue of 13.7% at 900°C. By contrast, the TGA plot of the pristine PIL nanoparticles shows a complete decomposition already at 600°C (Figure S2). The difference in mass residue between two samples determines a metallic Cu content of 9.6 wt.% in the PIL/Cu composite particles. This is in good agreement with our combustion‐based elemental analysis result of 10.1 wt% of Cu in the composite.

**FIGURE 4:**
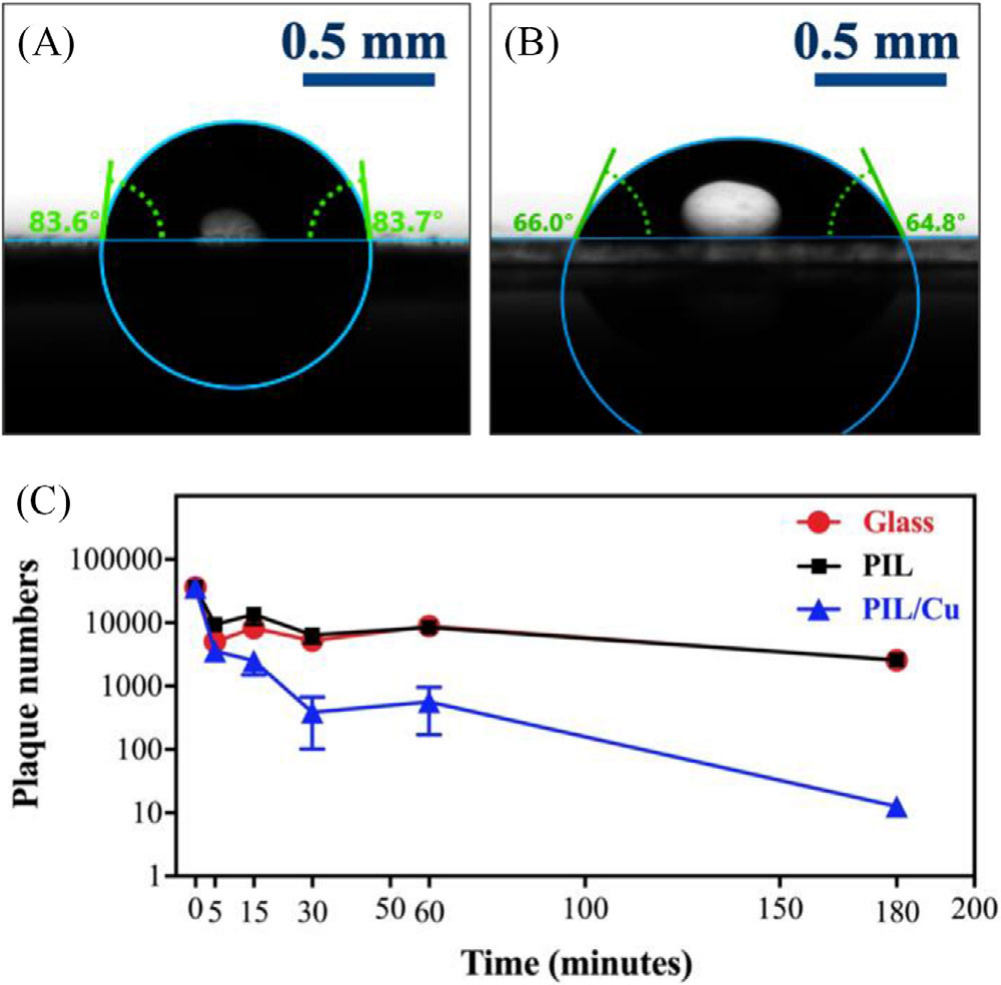
A, B), Water contact angle measurements of glass substrates coated with PIL nanoparticles, and the PIL/Cu composite nanoparticles, respectively. C), Characterization of antiviral activity of glass slides coated with PIL nanoparticles and the composite of the PIL/Cu nanoparticles against SARS‐CoV‐2 virions at various times

To explore the property of the coated surface with the as‐prepared dispersion of PIL/Cu colloidal nanoparticles, water contact angle measurements were performed (Figure 4A and 4B). The presence of Cu nanoparticles in the composite of PIL/Cu nanoparticles decreases the contact angle from 83.6° for pristine PIL nanoparticles to 65.4° for the composite, very possibly due to the increased surface hydrophilicity arising from the Cu nanoparticles.^[^ ^44^, ^45^ ^]^

The virucidal activity of glass substrates coated with pristine PIL, and the composite of PIL/Cu nanoparticles is shown in Figure 4C. Substrates were incubated with SARS‐CoV‐2‐containing solution for indicated times and residual infectivity was analysed by plaque assay. The pristine PIL nanoparticles coated on the glass substrate have little‐to‐no virucidal effect on SARS‐CoV‐2 virions in the first 60 minutes compared to uncoated glass. By contrast, the plot of virucidal activity of the composite of PIL/Cu nanoparticles indicates that within 30 minutes, 90% of SARS‐CoV‐2 virions is deactivated, and 180 minutes is enough for 99.5% deactivation. Thus, the introduced Cu nanoparticles play a decisive role in the antivirus function here. Cu nanoparticles have been a favorable choice as protective coating due to their known virucidal function. Yet, it is not fully clear here whether it is the Cu nanoparticles or the Cu^2+^ ions, or their joint effects that are responsible for the prominent virucidal effect of the PIL/Co composite coating. We will leave this discussion open and consider it as a potential future research topic. Note that the film coating sticks to the surface, unlike the majority of ethanol‐based liquid disinfectants that need to be repeatedly used to eliminate virus from surfaces. In this context, the colloidal dispersion of PIL/Cu composite nanoparticles is more effective in long‐term protection of surfaces against SARS‐CoV‐2 virions and helping reduce their spread through surface contact.

## 3 CONCLUSIONS

In conclusion, we presented the preparation of a composite film containing imidazolium‐based PIL nanoparticles with a dodecyl substituent and the Cu nanoparticles located on them. The unique quasi‐sphere multilamellar structure of PIL nanoparticles enables the water‐insoluble PIL to be well‐dispersed in water in the form of nanoparticles. In combination of the cationic charge on the PIL, the PIL nanoparticles can effectively stabilize Cu nanoparticles on their surface. The waterborne composite nanoparticles can spray onto and coat the substrate surface. The virucidal activity test proves such surface coatings have potential applications to deactivate SARS‐CoV‐2 virions from surfaces.

## Supporting information

Supporting Information

## Data Availability

Research data are not shared.
