# Supplementary material for: Colloidal dispersion of poly(ionic liquid)/Cu composite particles for protective surface coating against SAR‐CoV‐2
Source: Nano Sel. 2021 Jun 1;3(1):227–32. doi: 10.1002/nano.202100069 (PMC8242609; doi:10.1002/nano.202100069)
Supplement: Supplementary file 1 — Supporting Information [file NANO-3-227-s001.docx]

**Supporting Information**

**Colloidal dispersion of poly(ionic liquid)/Cu composite particles for protective surface coating against SAR-CoV-2**

Atefeh Khorsand Kheirabad^1†^, Xuefeng Pan^2†^, Siwen Long^3^, Zdravko Kochovski^2^, Shiqi Zhou^1^, Yan Lu^2,4^, Gerald McInerney^3^, Jiayin Yuan^1,*^

^1^Department of Materials and Environmental Chemistry (MMK), Stockholm University, 10691 Stockholm, Sweden. E-Mail: [jiayin.yuan@mmk.su.se](mailto:jiayin.yuan@mmk.su.se)

^2^Department for Electrochemical Energy Storage, Helmholtz-Zentrum Berlin für Materialien und Energie, Hahn-Meitner-Platz 1, Berlin 14109, Germany

^3^Department of Microbiology, Tumor and Cell Biology, Karolinska Institutet, 17177, Stockholm, Sweden.

^4^Institute of Chemistry, University of Potsdam, 14476 Potsdam, Germany

^†^ These two authors contribute equally to the manuscript.

**1. Experimental**
**1.1 Chemicals**

1-Vinylimidazole (99%) and copper (II) acetate monohydrate were purchased from Alfa Aesar. 1-Bromododecane was purchased from Acros Organic. 2,2'-Azobis[2-methyl-N-(2-hydroxyethyl)propionamide] (VA-086) was purchased from FUJIFILM Wako Chemicals. L-ascorbic acid and hydrazine hydrate 35 % solution in water were purchased from Sigma-Aldrich. All chemicals were used without any further purification. Solvents were of analytical grade.

**1.2 Instruments**

Nuclear magnetic resonance (NMR): ^1^H-NMR spectra were recorded at room temperature using a Bruker DPX-400 spectrometer operating at 400 MHz. CDCl_3_was used as a solvent for the measurement.

Scanning electron microscopy (SEM): The morphology of the samples was recorded on a JEOL 7000 operated at 3 kV. Samples were coated with a thin gold layer for 40 seconds before examination.

Transmission electron microscopy (TEM): The morphology of the nanoparticles was measured using a JEOL JEM-2100 transmission electron microscope (JEOL GmbH, Eching, Germany) operated at an acceleration voltage of 200 kV. Nanoparticles suspended in water were dried on continuous carbon-coated copper TEM grids (200 mesh, Science Services).

Cryogenic transmission electron microscopy (cryo-TEM): Cryo-EM specimens were prepared by applying a 4 μL drop of a dispersion sample to Lacey carbon-coated copper TEM grids (200 mesh, Science Services) and plunge-frozen into liquid ethane with an FEI vitrobot Mark IV set at 4 °C and 95% humidity. Vitrified grids were either transferred directly to the microscope cryogenic transfer holder (Gatan 914, Gatan, Munich, Germany) or stored in liquid nitrogen. Imaging was carried out at temperatures around 90 K. The TEM was operated at an acceleration voltage of 200 kV, and a defocus of the objective lens of about 2.5–3 μm was used to increase the contrast. Cryo-EM micrographs were recorded at a number of magnifications with a bottom-mounted 4 × 4 k CMOS camera (TemCam-F416, TVIPS, Gauting, Germany). The total electron dose in each micrograph was kept below 20 e^−^Å^−2^.

X-ray diffraction (XRD): XRD measurements were carried out using a Bruker D8 diffractometer in the locked coupled mode (2θ ranging from 10° to 80°) with Cu Kα1 radiation. The PIL/Cu composite sample was measured with an air-tight XRD holder from Bruker.

Thermal gravimetric analysis (TGA): TGA measurements were performed using a PerkinElmer (TGA 8000) from 25 to 900 °C under a constant argon flow (30 mL min^−1^) with a heating rate of 10 K min^−1^.

Contact angle measurement: The contact angle was measured using the image of a sessile drop with DI water at the points of intersection (three-phase contact points) between the drop contour and the projection of the surface (baseline) (KRÜSS instruments, DSA25E, CA).

**1.3 Synthesis of the ionic liquid monomer (3-dodecyl-1-vinylimdiazolium bromide)**

9.4 g of 1-vinylimidazole and 25g of 1-bromododecane (1:1 molar ratio) and 20 ml of ethanol were mixed together inside a 100 ml round bottom flask at 40 ^o^C for 16 h. A needle was used to reduce the pressure inside the flask. In the next day, the reaction mixture was added drop by drop to 1L diethyl ether and the precipitate was filtered off gently, and dried at 40 ^o^C using vacuum oven.

**1.4 Synthesis of poly(1-*n*-dodecyl-3-vinylimidazolium bromide) nanoparticle dispersion**

5g of monomer and 150 mg of a water-soluble thermal initiator VA-086 were added to 100 ml of water inside a 250ml round bottom Schlenk flask. The flask was treated with three freeze-pump-thaw cycles. The reaction was stirred at 70°C for 16 h. Next day, the polymer dispersion was filtered off using a filter paper and a funnel to remove impurities and big aggregation of nanoparticles. The proton nuclear magnetic resonance (^1^H-NMR) spectra is shown in Figure S1, where the chemical shifts of all signals are well-assigned to individual protons in polymer.

**1.5 Preparation of the PIL/Cu colloidal nanoparticle dispersion**

2 ml of the PIL nanoparticle dispersion was diluted with 9 ml of water. Then 1 ml of water containing 40 mg of copper acetate salt was added to the solution. After stirring for 2 h, aqueous hydrazine solution (35 wt.%) was dropwise added to the solution. Very gentle change of color happened during this step. The solution was sonicated for ca. 20 min. Then, the solution was placed in an oil bath under stirring at 80°C for overnight. The next day, the color of the solution changed to pink. Next, the solution was sonicated again for ca. 20 min. In the following, by using a 5 μm filter and a syringe the solution was filtered off to remove any big aggregates.

**1.6 Coating procedure**

A glass slip with size of 24 × 24 mm and thickness of 0.5 mm was treated with an oxygen Plasma instrument for 3 min to make the substrate surface hydrophilic and clean. At the final stage, with help of a spray coating machine (a spray machine with pore size of 100-micron meters, a flow rate of 2 ml per min, and a point size of 6.5 cm × 6.5 cm at a distance of 8.5 cm), the hydrophilic surface of glass slip was coated with the PIL/Cu nanoparticles dispersion for ca. 30 seconds with a film thickness of *ca.* 100-micron meters. The rinsed film was dried under vacuum (5 × 10^-2^ mbar) at 80 ^o^C overnight.

**1.7 Antiviral activity tests of hybrid coating**

Cover glasses were placed into a 6 cm dish and 50 μL containing a known number of infectious units of SARS-CoV-2 was applied in the centre of the glass. The droplet was immediately covered by uncoated cover glass to spread the virus on the whole area. After incubation at specified time, usually 5, 15, 30, 60 and 180 min, 1950 μL 1X PBS was added to the coverslips, the top coverslip was lifted and the virus-exposed sides of both coverslips were washed with PBS by pipetting 3 times to collect the virus species. All washes were collected and used for titre determination by plaque assay. Briefly, Vero E6 cells (ATCC-CRL-1586), maintained in Dulbecco’s modified Eagle medium (Gibco) supplemented with 10% fetal calf serum and 1% penicillin–streptomycin and cultured at 37 °C in a humidified incubator with 5% CO_2_, were washed with PBS, and two-fold serial virus dilutions of SARS-CoV-2 was added in 200 μL minimal essential medium (Invitrogen) supplemented with 0.2% bovine serum albumin, 2 mM L-glutamine, and 20 mM HEPES with periodic shaking for 1 h 37°C. Virus solutions were then removed and cells washed with PBS before addition of 1 mL of prewarmed overlay (2% Methylcellulose: propagation media containing 2% FBS= 2:3). At 48-72 h post infection, cells were fixed with 4% formaldehyde and stained with crystal violet solution after removal of the overlay and plaques were manually quantified. The determination of all virus titres was performed in triplicate.

**2. Supplementary Data**

**2.1 ^1^H-NMR spectrum of PIL**


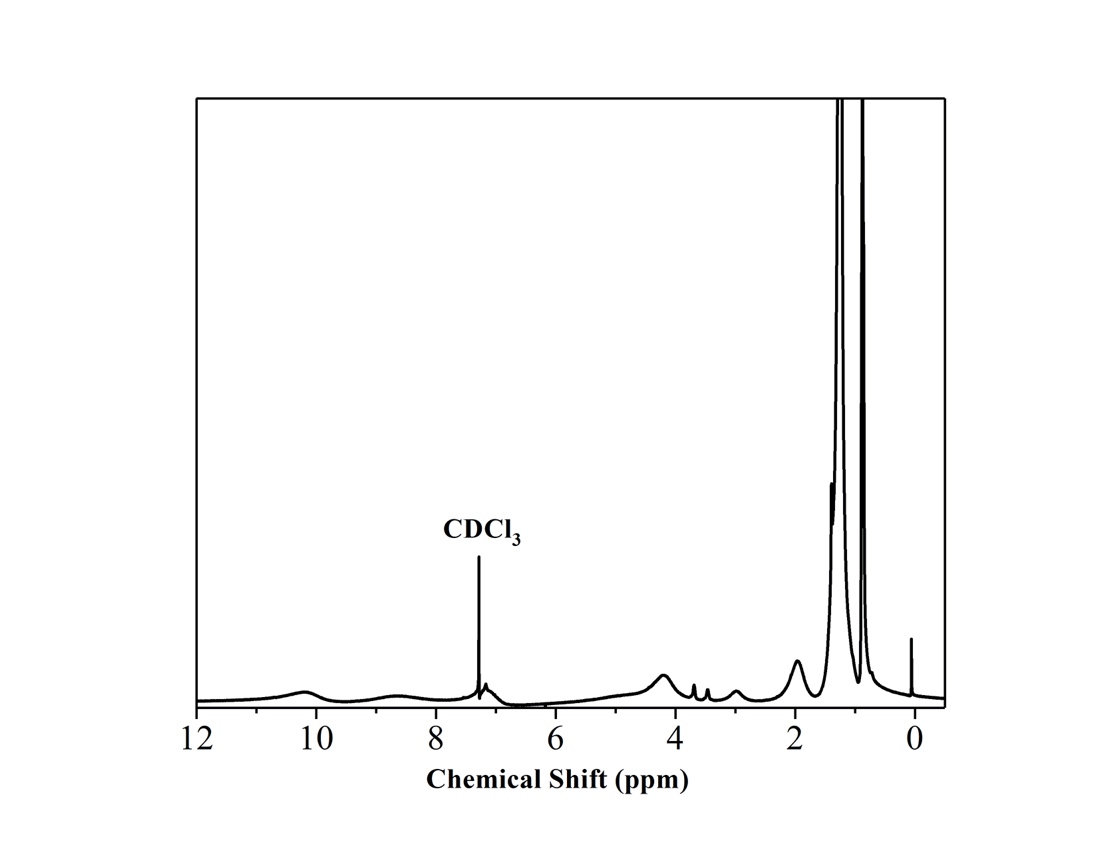


**Figure S1.** ^1^H-NMR spectra of poly(3-dodecyl-1-vinylimdiazolium bromide) in CDCl_3_.

**2.2 TGA analysis of PIL nanoparticles**

**
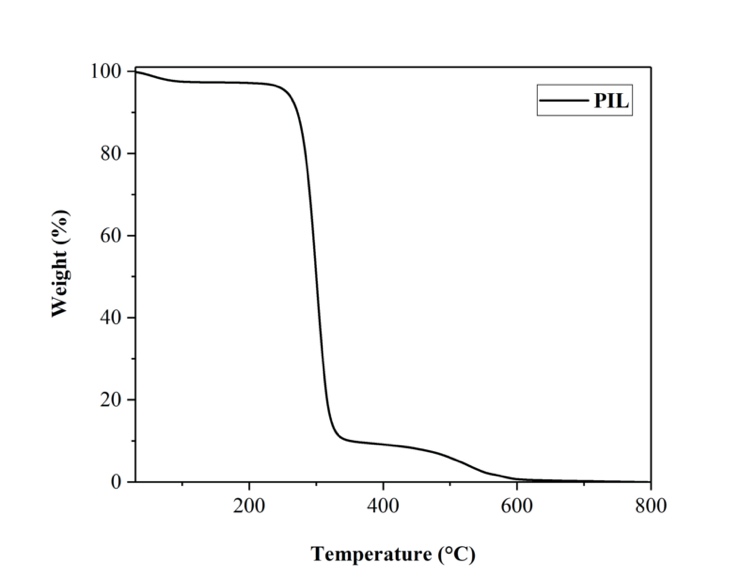
**

**Figure S2.** TGA plot of PIL nanoparticles under air from room temperature to 900 ^o^C
